# Toscana virus infection acquired in Campania, Italy, in a Hungarian traveller: case report with mini-review

**DOI:** 10.1186/s40794-026-00306-2

**Published:** 2026-05-20

**Authors:** Mohamed Mahdi, Imre Bakos, Anita Koroknai, Márk Kozák, Nikolett Csonka, Anna Nagy, István Zsolt Várkonyi

**Affiliations:** 1 Department of Infectology, Faculty of Medicine, University of Debrecen, Debrecen, 4031 Hungary; 2 National Reference Laboratory for Viral Zoonoses, National Center for Public Health and Pharmacy, Budapest, 1097 Hungary

**Keywords:** Toscana virus, Phlebovirus, Meningitis, Naples, Travel medicine, Lineage A

## Abstract

**Background:**

Toscana virus (TOSV), a sandfly-borne phlebovirus, is a leading cause of viral meningitis in the Mediterranean region and central Italy. Despite its prominence in endemic areas, TOSV infection is frequently underdiagnosed, particularly among international travellers.

**Case presentation:**

A 24-year-old Hungarian male patient developed acute serous meningitis two weeks after returning from Naples, Italy. Symptoms began with a severe headache, accompanied by two episodes of vomiting. Cerebrospinal fluid (CSF) analysis showed lymphocytic pleocytosis, elevated protein, and normal glucose levels. TOSV RNA was detected in CSF by RT-PCR, and a 350-nucleotide S-segment sequence showed 99% nucleotide identity to a TOSV lineage A strain previously identified in Italy. Seropositivity in the convalescent serum sample also confirmed the acute infection. Interestingly, blood panel tests showed markedly elevated total IgE levels (437 kU/L).

**Conclusion:**

To our knowledge, this represents the first molecularly confirmed case of TOSV neuroinvasive disease acquired in the Campania region since 2005. This report emphasizes TOSV as an emerging travel-associated neurotropic pathogen beyond its traditional central Italian focus, and supports the inclusion of TOSV in diagnostic panels for travellers with acute neurological symptoms following exposure in Mediterranean regions.

## Introduction

Toscana virus (TOSV; *Phlebovirus toscanaense*) is a member of the *Phlebovirus* genus within the *Phenuiviridae* family, *Hareavirales* order and the class *Bunyaviricetes*, and was first isolated in 1971 from sandflies collected in the Tuscany region of Italy [1–4]. TOSV is neurotropic, and ranks among the leading causes of aseptic meningitis and other neuroinvasive diseases during the summer months in endemic Mediterranean areas, alongside enteroviruses and herpesviruses [5]. So far, three main genetic lineages of TOSV have been identified: Lineage A predominates in Italy (especially central and northern regions), and has also been reported in France, Türkiye, Tunisia, and Algeria; Lineage B circulates in Portugal, Spain, France, Morocco, and parts of Türkiye and Croatia; and Lineage C has been detected in Croatia and Greece. Co-circulation of multiple lineages occurs in overlapping geographic areas, such as France, Türkiye, and Croatia. However, no clear association has been established between lineage and disease severity or specific clinical manifestations [6].

### Epidemiology

TOSV is well-documented in central Italy (Tuscany, Marche, Lazio), with hundreds of cases confirmed and reported annually [4, 7]. In Southern Italy, the Campania region, including Naples, has high sandfly density [8, 9]. As of the date of writing this manuscript, the Italian National Institute of Health (ISS / EpiCentro) national arboviral surveillance reported 113 cases of TOSV infections (112 autochthonous cases and 1 case associated with travel abroad, with one death) [10]. Previously, a molecular survey of CNS infections in southern Italy (including Naples/Campania) found Toscana virus RNA in 5.6% of CSF samples from patients with meningitis/encephalitis in the summer season of 2005 [11].

In Italy, a 10-year review of arboviral epidemiology found TOSV to be a major endemic threat alongside West Nile virus, with rising cases linked to vector ecology changes [12]. Moreover, national surveillance analyses reports a 2.6-fold increase in neuroinvasive cases caused by TOSV in Italy during 2022–2023 (276 cases), compared to 2016–2021 (a total of 607 cases), with cases mostly concentrated in northern/central regions like Emilia-Romagna [7].

TOSV IgG seroprevalence ranged from 22.95% (2003–2004) to 26.75% (2013–2014) in central Italy (Siena, Tuscany), whereas southern Italy (Bari, Apulia) showed consistently low rates of 2.90% (2004) and 1.85% (2015), according to a longitudinal serosurvey spanning over a decade [13].

Beyond Italy, TOSV circulates across the Mediterranean basin and parts of North Africa. In the broader Mediterranean region, historical seroprevalence data indicate rates of 0–23.5% in Greece, with the highest values observed in the Greek islands [14, 15]; 37.5% in Croatia, predominantly among residents of coastal and island areas [16]; and 9.5% in Tunisia [17]. In Hungary, no autochthonous TOSV cases have been documented to date, with all reported infections classified as imported.

The first imported case of TOSV infection in Hungary was documented in 2019. To date, a total of four such imported cases have been confirmed, with the patient described in this manuscript representing the fifth case. All patients had travelled to the Mediterranean region prior to symptom onset (2019: Malta; 2022: Croatia and Italy; 2022: Croatia; and 2024: Malta) and exhibited neurological manifestations according to data from the Hungarian National Centre for Public Health and Pharmacy.

A literature search revealed no previously published molecularly confirmed Toscana virus infections acquired in the Naples area in Hungarian travellers; therefore, this case appears to be the first documented in recent years. Our report is further strengthened by partial sequencing of segment S, which enabled lineage identification. Notably, the clinical course was characterized by the absence of fever and an unexpected, marked elevation in IgE levels. These unusual features raise important questions regarding the underlying pathogenic mechanisms of TOSV infection and highlight the need for further research into its immunological and virological behaviour.

### Clinical and diagnostic considerations

Routine laboratory parameters of TOSV infection are generally non-specific, and often fail to provide a definitive diagnosis. Peripheral blood counts may remain within normal limits; however, mild leukocytosis or leukopenia, as well as thrombocytopenia, have been documented in confirmed TOSV encephalitis cases. Importantly, inflammatory markers such as C-reactive protein (CRP) are typically normal or only minimally elevated, consistent with the predominantly viral and non-pyogenic nature of TOSV-induced central nervous system inflammation. This pattern underscores the need for virus-specific diagnostic testing, as standard inflammatory profiles may appear deceptively unremarkable, even in the presence of active neuroinfection [18]. CSF analysis is essential in the diagnostic workup of TOSV encephalitis. Findings typically include lymphocytic pleocytosis (30–900 cells/µL), elevated protein levels, and normal glucose concentrations. This pattern is consistent with viral neuroinfections, and in the appropriate epidemiological context, should raise suspicion for infection with TOSV [19].

Diagnosis of TOSV infection relies on molecular and serological methods, as clinical presentation overlaps with other viral CNS infections [20]. During the acute phase, viral RNA can be detected in CSF, and less frequently, in the blood using real-time RT-PCR, the current gold standard [20]. Virus isolation in cell culture is possible but not routinely performed. The viraemia is short, lasting about 5 days, and viral RNA is detectable in serum or CSF approximately 2–7 days post-exposure. Therefore, early sampling is crucial for the application of molecular diagnostic assays [21–23].

Recent studies have also demonstrated that urine may serve as an alternative clinical specimen for TOSV RNA detection, especially when CSF or blood samples are unavailable or collected outside the optimal viremic phase [24]. In addition, metagenomic next-generation sequencing (mNGS) has proven useful in identifying TOSV RNA in challenging diagnostic cases, particularly when targeted molecular assays such as real-time RT-PCR yield negative or inconclusive results despite clinical suspicion, or when multiple pathogens are suspected [25].

Serological testing plays a key role, particularly when molecular assays are negative or unavailable. Detection of TOSV-specific IgM and IgG antibodies is commonly performed using indirect immunofluorescence (IIFA), ELISA, or immunochromatographic assays. IgM indicates recent or acute infection, while IgG reflects past exposure or immune memory. The humoral response typically develops early, with both antibody classes detectable around the time of hospital admission. IgM levels usually decline within a few months, but may persist for up to 6–20 months. IgG antibodies remain detectable long-term, independent of age, sex, or disease severity [6]. Paired serum samples collected in the acute and convalescent phases are recommended to confirm seroconversion or a four-fold rise in IgG titres, in line with ECDC guidelines for the serological diagnosis of acute TOSV infection [20].

Given the possibility of cross-reactivity with antigenically related phleboviruses (such as Naples, Sicilian, and Punique), confirmatory testing with neutralization assays is advised when diagnostic specificity is critical.

In clinical settings, serology complements PCR, and is particularly valuable in the later stages of infection, when viral RNA may no longer be detectable [5].

Neuroimaging is useful in the evaluation of TOSV encephalitis, especially in patients presenting with central nervous system involvement, primarily to exclude alternative causes and assess complications. Head CT scans are frequently normal or show only non-specific findings. Brain MRI is more sensitive, but often unremarkable. When abnormal, it may show rare, non-specific T2-weighted and FLAIR hyperintensities in the thalami, basal ganglia, brainstem, or cerebellum. These changes are not pathognomonic, but may support clinical suspicion of TOSV encephalitis in the appropriate epidemiological context and guide further diagnostic workup [19, 26].

### Treatment and prognosis of infection

To date, no specific antiviral therapy or vaccine is currently available for TOSV infection. Management is primarily supportive and symptomatic, focusing on alleviating symptoms and preventing complications. In mild cases, presenting primarily with fever, headache, and myalgia, outpatient care with analgesics, antipyretics, and adequate hydration is typically sufficient. When signs of neuroinvasion are present; such as meningitis, encephalitis, or meningoencephalitis, hospitalization is often required for close monitoring and supportive interventions [27]. Severe manifestations, such as seizures or altered mental status, may necessitate intensive care unit admission, anticonvulsant therapy, and mechanical ventilation in rare instances of respiratory compromise [6, 11, 19]. Corticosteroids have been used empirically in some cases of severe encephalitis, though evidence for their efficacy is limited [28]. Vector control and personal protective measures remain the cornerstone of prevention, particularly for travellers to endemic areas during peak sandfly season (June–September).

The prognosis for TOSV infection is generally favourable, with the majority of cases resolving spontaneously within days to weeks [19]. In neuroinvasive forms, which predominate in reported cases, over 95% of patients achieve complete recovery, particularly among immunocompetent individuals under 65 years of age. However, a subset may experience persistent sequelae, including sensorineural hearing loss [18, 29], cognitive impairments [30], or mild neurological deficits such as ataxia or paresis [31]. Mortality is exceptionally rare, and to our knowledge, no evidence supports more than 6 confirmed fatalities as of January 2026 [19], with no deaths noted in recent Hungarian imported cases.

## Case presentation

A previously healthy 24-year-old Hungarian male presented to the emergency department on September 13, 2025, with a two-day history of severe nuchal headache (8/10 intensity) and two episodes of vomiting. Symptoms began 12 days after returning from a one-week vacation in Naples, Italy (August 25–September 1, 2025), where he engaged in outdoor activities without using insect repellents.

Seven days prior to admission, he was canoeing in Tiszafüred, Hungary, where he noted a mosquito bite; however, he did not recall any sandfly exposure in either Hungary or Italy. On examination, he was afebrile, with blood pressure of 146/95 mmHg, no nuchal rigidity, and no focal neurological deficits. Double vision, photophobia, speech, or swallowing difficulties were absent. Native head CT scan was normal. He received Dexibuprofen and Metamizole with partial relief of symptoms, and was admitted to the Infectious Diseases ward on September 14.

On the day of admission, CSF analysis revealed pleocytosis with a white blood cell count of 192/µL (80% mononuclear cells, comprising 48% macrophages and 28% lymphocytes; 20% polymorphonuclear cells), markedly elevated total protein of 1179 mg/L (reference: 150–450 mg/L) and albumin of 792 mg/L (reference: 100–300 mg/L), and glucose of 3.1 mmol/L (serum glucose: 6.2 mmol/L, yielding a normal CSF-to-serum ratio) (Table 1).

**Table 1:** Summary of blood test results and CSF analysis

Parameter	Result	Reference / Notes
**CSF Analysis**		
WBC count	192 /µL	Pleocytosis
Mononuclear cells	80%	48% macrophages, 28% lymphocytes
Polymorphonuclear cells	20%	–
Total protein	1179 mg/L	Reference: 150–450 mg/L
Albumin	792 mg/L	Reference: 100–300 mg/L
Glucose	3.1 mmol/L	Serum glucose: 6.2 mmol/L (CSF-to-serum ratio normal)
**Blood Tests**		
Leukocyte count	13.42 × 10⁹/L	Neutrophils 84.3%
C-reactive protein (CRP)	6.32 mg/L	Reference: < 5
Creatine kinase (CK)	359 U/L	Reference: < 200 U/L
Lactate dehydrogenase (LDH)	294 U/L	Reference: < 250 U/L

Multiplex PCR was performed by the Department of Medical Microbiology at the University of Debrecen, using the BIOFIRE FILMARRAY Meningitis/Encephalitis (ME) Panel (BioMérieux Hungária Kft., Budapest, Hungary), following the manufacturer’s instructions. This panel detects 14 targets, including Cytomegalovirus (CMV), Enterovirus (EV), Herpes simplex virus 1 (HSV-1), Herpes simplex virus 2 (HSV-2), Human herpesvirus 6 (HHV-6), Human parechovirus (HPeV), Varicella zoster virus (VZV), Escherichia coli K1, Haemophilus influenzae, Listeria monocytogenes, Neisseria meningitidis, Streptococcus agalactiae, Streptococcus pneumoniae, and Cryptococcus species (C. neoformans/C. gattii). Blood tests on September 13 demonstrated leukocytosis of 13.42 × 10⁹/L (84.3% neutrophils), CRP of 6.32 mg/L, creatine kinase of 359 U/L, and lactate dehydrogenase of 294 U/L. The patient received supportive management with intravenous fluids and analgesics, resulting in the resolution of headache and vomiting within 48 h.

**Fig. 1:**
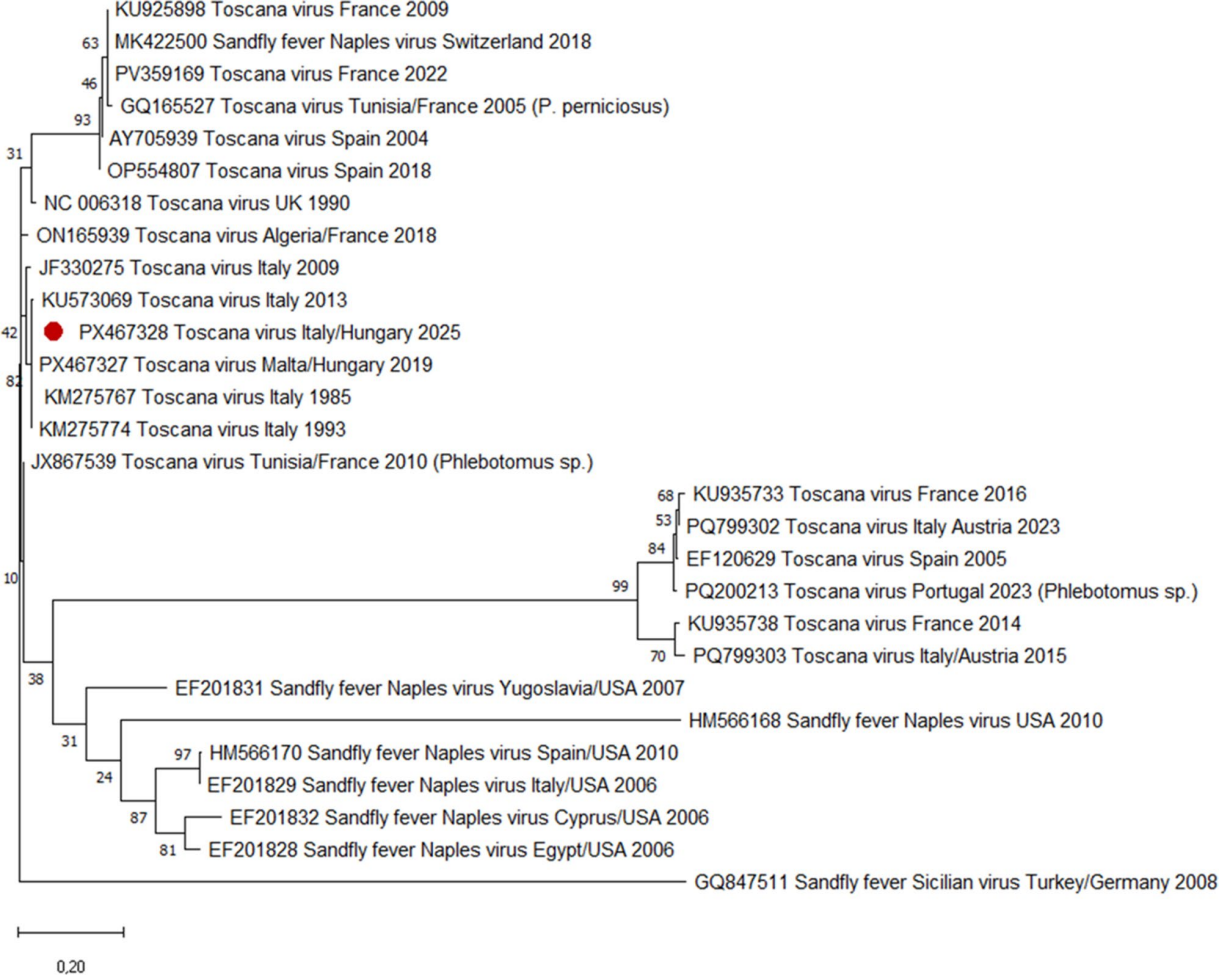
Phylogenetic analysis of Toscana virus (TOSV) sequences based on a 350-nucleotide region of the S-segment nucleoprotein gene. A maximum-likelihood phylogenetic tree was generated in MEGA 11 (Molecular Evolutionary Genetics Analysis software, version 11) using ClustalW alignments. Bootstrap analysis with 1,000 replicates was performed to assess the reliability of the tree topology. Evolutionary distances were calculated using the Kimura 2-parameter model (Gamma-distributed with invariant sites). Sandfly fever Sicilian virus was used as the outgroup. The sequence obtained from the CSF sample of the Hungarian patient is indicated by a red circle (GenBank accession number: PX467328). The Hungarian sequence clusters with previously characterized TOSV lineage A reference strains, and shows high nucleotide sequence identity to these sequences; it was therefore provisionally assigned to lineage A. However, consistent lineage annotation for all sequences in the tree was not feasible due to incomplete metadata in public databases.TOSV sequences were labelled as follows: GenBank accession number, strain name, country of import/country of diagnosis, and year of isolation/submission (if the data were available). For non-human sequences, the host species is indicated in brackets

Sandfly fever virus serology on September 16 was positive for TOSV IgM, IgA, and IgG in serum and CSF samples, with an increasing IgG titer in paired sera (1:320 in sample I. and 1:3200 in sample II.) as measured by the Sandfly fever virus Mosaic 1 IIFT test (Euroimmun Medizinische Labordiagnostika, Lübeck, Germany). Serologic tests were negative for Tick-borne encephalitis virus, West-Nile virus (Flavivirus Mosaic IIFT; Euroimmun Medizinische Labordiagnostika, Lübeck, Germany), and Lymphocytic choriomeningitis virus (in-house IIFA, performed as described previously [32]). Parallel real-time RT-PCR and S-segment-specific nested RT-PCR assays were performed for the detection of TOSV, revealing viral RNA in the patient’s CSF (Ct value: 29.59), while the serum sample remained negative. The TaqMan assay utilized primer sets previously described by Perez-Ruiz et al. [22], and was conducted on a LightCycler 2.0 instrument (Roche Life Science, Basel, Switzerland) using the LightCycler^®^ TaqMan^®^ Master kit (Roche Life Science, Basel, Switzerland). Nested RT-PCR was performed as previously described by Charrel et al. [33], with minor modifications to accommodate our in-house enzyme system and PCR platform. Partial S-segment sequencing (350 nucleotides, nucleoprotein gene) showed a 99% nucleotide identity to TOSV strains from Italy (GenBank accession numbers: KM275767; KM275774), isolated in Firenze in 1985, and Macerata in 1993, indicating that the virus is most consistent with lineage A (Fig. 1). Another shorter sequence from a Hungarian patient, who travelled to Malta in 2019 (GenBank accession number: PX467327), also showed a high degree of nucleotide similarity to our sequence.

An immune profile on September 24 showed elevated total IgE at 437 kU/L (reference: <100 kU/L, > 4× upper limit) without eosinophilia or history of atopy or allergy. The patient was discharged on September 17 in good condition. At follow-up on September 24, he reported transient orthostatic dizziness, fatigue, tinnitus, and brief stabbing headaches (rated 1/10), all resolving with rest and fluid intake. Ear, nose, and throat evaluation was recommended but not pursued by the patient.

By the final follow-up on October 3 2025, he was asymptomatic with a normal neurological examination and blood pressure of 146/95 mmHg, with complete symptom resolution.

## Discussion

To our knowledge, this is the first PCR-confirmed neuroinvasive TOSV infection acquired by a Hungarian traveller in the Campania region (specifically Naples), although it is not the first PCR-confirmed imported TOSV case among Hungarian patients overall. While TOSV has been reported to be endemic in central Italy, and IgG seroprevalence rates were consistently reported between 22.95% (2003–2004) and 26.75% (2013–2014) in Siena, Tuscany [7, 12, 13], to our knowledge, no dedicated seroprevalence data are available for the Campania region, including Naples. Despite clinical reports of neuroinvasive TOSV infections in Campania since 2005 [11], population-based serological surveys remain absent for this densely populated southern Italian area. This gap limits precise assessment of TOSV exposure in a region with favorable sandfly habitats and high vector contact potential, underscoring the need for targeted sero-epidemiological studies.

The patient did not travel to Tuscany or Marche, and the incubation period (12–14 days) aligns well with sandfly exposure in Naples, even though the patient does not recall the sandfly bite. The 350-nt TOSV sequence from CSF was near identical to reference strains of Lineage A from Italy (GenBank accession numbers: KM275767; KM275774), isolated in Firenze in 1985, and Macerata in 1993, suggesting the presence of TOSV strains related to the central Italian genotype, although this inference is limited by the short sequence length.

The overlapping genomic regions shared 99% nucleotide sequence identity and encoded identical amino acid sequences, indicating a high degree of similarity. However, given the relatively short length of the sequence analysed, this observation should be interpreted with caution. The similarity between the sequence detected in this report and the previously reported 2019 Hungarian sequence may reflect the circulation or repeated introduction of closely related virus strains in the region, or alternatively, a relatively low evolutionary rate within this genomic segment. However, the limited sequence data preclude definitive conclusions regarding lineage assignment, viral persistence, transmission pathways, or geographic origin, and more extensive genomic data would be required to clarify these aspects.

Interestingly, the complete absence of fever in this case of TOSV infection is striking and clinically significant, as pyrexia is a prominent feature in reported neuroinvasive infections, occurring in 76–97% of patients across large series [19, 34]. Here, despite clear evidence of acute aseptic meningitis, the patient remained afebrile throughout the illness, with no documented temperature elevation in the history, on presentation, or during hospitalization. This afebrile onset with immediate progression to symptomatic meningitis underscores the expanding phenotypic spectrum of TOSV beyond the classic febrile “pappataci fever” or febrile meningitis syndromes typically described. Such presentations may evade early clinical suspicion, particularly in returning travellers from endemic regions such as Naples, Italy, especially during summer peaks.

Notably, total IgE was 437 kU/L; the highest value identified after a review of the available literature on TOSV infections. Sandfly saliva contains potent allergens, and IgE-mediated responses may amplify neuroinflammation [35]. While speculative, this finding warrants further study in larger cohorts. Moreover, while some viral infections are known to trigger increases in IL-4, and IL-13; among other cytokines, which in turn activate Th2 cell responses leading to increased IgE levels, so far, this has not been described for TOSV, and unfortunately, we were not able to complement our tests to assay for IL levels.

This case has public health implications. TOSV should be considered in all summer meningitis cases post-travel to coastal Italy, not just Tuscany, as well as after travel to other endemic Mediterranean areas. Molecular and serological diagnostics are essential, as TOSV is not included in standard test panels, especially in non-endemic countries.

While sandfly vectors are present in Hungary with potential expansion, the absence of detected TOSV circulation and lack of local cases make autochthonous acquisition unlikely; imported infection from Naples remains the most likely explanation in our case.

## Conclusion

We hereby describe the first PCR-confirmed case of Toscana virus meningitis acquired in Naples, Italy, with partial genomic sequence findings consistent with a virus related to previously described lineage A strains, in a Hungarian patient returning from a summer vacation. This case extends the recognized endemic range of TOSV beyond central Italy and highlights its role as an emerging travel-related neurotropic pathogen. Notably, the complete absence of fever despite clear neuroinvasive disease expands the clinical phenotype, emphasizing the need for TOSV testing in afebrile summer meningitis following Mediterranean exposure. Moreover, marked total IgE elevation raises the possibility of a sandfly saliva-driven or virus-induced Th2 immune response, warranting cytokine profiling and validation in larger cohorts to elucidate underlying mechanisms.

## Data Availability

No datasets were generated or analysed during the current study.
